# Camp Life for Medical Students

**Published:** 1910-08-27

**Authors:** 


### The Hospital

A Journal of

The Medical Sciences and Hospital Administration.

New Series. No. 185, Vol. VII. [No. 1257, Vol. XLVIII.] SATURDAY, AUGUST 27, 1910.

### CAMP LIFE FOR MEDICAL STUDENTS.

The medical student in the twentieth century does not take, as a rule, an extravagantly large allowance of holidays, for the curriculum is so extensive that to qualify within a reasonable number of years absorbs his energies during the greater part of each year of his long training. It is therefore the more necessary that such holidays as he does take shall reinvigorate and refresh him for the ensuing period of study. Precisely how and where he shall thus spend his vacations depends very largely upon individual tastes and opportunities; but it is perhaps permissible to point out, if not to urge, the great benefit of occupying a week or two under canvas in one or other of the Auxiliary Forces of the Crown. Such a recreation has many things to recommend it. First and foremost it provides a holiday of the most health-giving and rejuvenating type. It combines fresh air and sunshine with early rising and hard physical labour, and as a contrast to the enervating grind of medical study, whether in the wards, dissecting-rooms, or laboratories, nothing could be more complete. It might almost without exaggeration be said that if from a medical school are picked out those who belong to some unit of the Territorial Force and train with it in camp, the group will include the most strenuous and promising students and those most likely to reflect credit on their school in their subsequent career. The discipline of military service as it is nowadays understood is a moral tonic of the most wholesome nature, and the character-forming influence of camp life during student days has proved an asset of no small magnitude to many a practising medical man.

In the last issue (The Hospital, August 20, 1910, p. 613) were reproduced some photographs taken during the recently concluded camp at Aldershot of the medical branch of the Officers' Training Corps. It is because the training season is just concluded that the subject is now especially worth the attention of all medical students; for this reason, that the autumn is the best time for recruits to join any unit, be it horse, foot, or artillery, of the Territorial Force, as then sufficient preliminary drill and instruction can be obtained to render the novice reasonably efficient before the next summer training season comes round. To all medical students not already enlisted, except those disqualified by actual physical disability, we would say "Go and join the ranks of the Territorials: you will have to give up a week or a fortnight of your well-deserved and possibly scanty holiday, but you will never regret it." There is no need to discriminate between the different arms of the Service. To many the attractions of a field ambulance will be the greatest, especially to those who have begun ward work; but it is probable that a greater change and a more excellent result can be obtained by joining an infantry battalion, an artillery battery, or, best of all, a yeomanry regiment. At any rate, the student should certainly join—at first anyhow—in the ranks, whatever branch of the service is selected. He will thus learn lessons of inestimable value in his after life, and learn them all the more thoroughly in that the teaching will be imperceptible and the process of learning quite involuntary.

Hitherto the benefit to the individual has been chiefly insisted upon. There is, of course, another aspect of the question, and one which cannot be overlooked. As one of the body politic, the qualified medical man has peculiar claims upon the indulgence of the State in matters of social service, claims which are recognised in many conscript countries by the exemption of medical students from compulsory military service. For the medical man is by virtue of his professional knowledge and training competent to render valuable service to his country in time of national emergency. He can transform himself into one of the *personnel* of an indispensable part of any army—its medical service—almost without any special training at all. It is, therefore, open to a medical student to argue that, since he is on the way to becoming a qualified medical man, his obligation to bear any further share of the burden of preparation for national defence does not exist. Such an excuse for the avoidance of the patriotic duty of learning to take part in national defence cannot be admitted as valid. For no one can tell how or when that crisis will arise which it is the object of all schemes of national defence to avert or to meet. All that is fairly certain is that when it does come it will come suddenly; and the medical student is not relieved of the responsibilities of citizenship because he is intended for a profession which has a unique method of discharging them. A medical man should be, as the name implies, both a doctor and a man; and there is no more manly pursuit than that of arms, and none more calculated to develop those qualities of resource and decision which are pre-eminently requisite for the successful doctor. Therefore, let the duties of a man rest upon the medical student equally with his contemporaries in other walks of life, let him accept them and do his very best to carry them out in such a way as shall bring most credit to himself and honour upon the profession into which he hopes to enter.

One more point in conclusion. In after years the regimental surgeoncy of the neighbouring unit may be offered to, or coveted by, the practitioner; or he may accept a commission in a field ambulance or a general hospital of the Territorial Force. The knowledge of drill, discipline, and of the routine generally of a camp, which he who himself has been through the ranks attains, is of peculiar value to such an officer, and renders him much more at home amid the surroundings of a camp.

Thus the prophylactic, as well as the curative, treatment of blistered heels or saddle-galled buttocks are matters which he who has himself suffered from either of those particularly painful afflictions is far more likely to possess than is he who has never served his time in the ranks. We would, therefore, have every student cross-examine himself on this subject of military service; and, unless he can justify his abstention, make up his mind to join this autumn such a unit as the circumstances of his case render most accessible or most desirable.

